# Innate olfactory preferences for flowers matching proboscis length ensure optimal energy gain in a hawkmoth

**DOI:** 10.1038/ncomms11644

**Published:** 2016-05-13

**Authors:** Alexander Haverkamp, Julia Bing, Elisa Badeke, Bill S. Hansson, Markus Knaden

**Affiliations:** 1 Department of Evolutionary Neuroethology, Max Planck Institute for Chemical Ecology, Hans-Knöll Straße 8, D-07745 Jena, Germany

## Abstract

Cost efficient foraging is of especial importance for animals like hawkmoths or hummingbirds that are feeding ‘on the wing', making their foraging energetically demanding. The economic decisions made by these animals have a strong influence on the plants they pollinate and floral volatiles are often guiding these decisions. Here we show that the hawkmoth *Manduca sexta* exhibits an innate preference for volatiles of those *Nicotiana* flowers, which match the length of the moth's proboscis. This preference becomes apparent already at the initial inflight encounter, with the odour plume. Free-flight respiration analyses combined with nectar calorimetry revealed a significant caloric gain per invested flight energy only for preferred—matching—flowers. Our data therefore support Darwin's initial hypothesis on the coevolution of flower length and moth proboscis. We demonstrate that this interaction is mediated by an adaptive and hardwired olfactory preference of the moth for flowers offering the highest net-energy reward.

## Abstract (web summary)

Foraging is energetically demanding for animals like hawkmoths that feed while flying. Here, Haverkamp *et al*. show that *Manduca sexta* has an innate preference for feeding on species of *Nicotiana* whose flower corolla length best matches the length of their proboscis, which allowed more efficient foraging and yielded the highest caloric gain.

## Graphical abstract

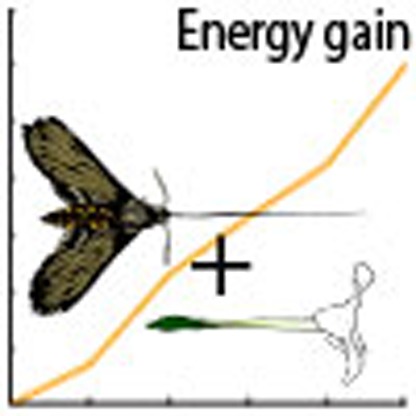

When foraging, pollinators have to balance their energy expenditure against their energy gain to maximize their reproductive success^1^,^2^. Through these energetic economic decisions pollinators are not only influencing their own ecology, but do also alter their evolutionary relationship with the flowers they pollinate^3^. Darwin was among the first to propose a coevolution between pollinators and flowers, based on pollen transfer and increased nectar uptake^4^. On the basis of his initial observation that red clover flowers can only be pollinated by long-tongued bumblebees, Darwin suggested that the morphology of pollinators and flowers has coevolved ‘ […] in the most perfect manner'. From this initial hypothesis, it has been argued that pollinators would forage most effectively on flowers matching their morphological, as well as their sensory requirements. Thereby pollinators would drive the evolution of floral traits, leading to the development of ‘pollination syndromes', where phylogenetically unrelated flowers match the preference of their pollinators in form, colour and scent^4^,^5^,^6^. However, long-tongued hawkmoths and other pollinators with a specialized morphology have frequently been observed foraging on flowers not matching their morphological traits^7^,^8^. This generalization among pollinators raises the question how specialized pollination systems can evolve and persist.

Floral volatiles have been argued to inform a pollinator about the potential energy gain to be obtained from a flower, allowing the pollinator to recognise a most rewarding flower among others and thereby facilitating a coevolution of plants and pollinators^9^. Pollinators are faced with the dilemma that they cannot infer directly about the nectar amount of a certain flower^10^ and even less about the specific foraging costs associated with that flower. Hence, pollinators have to rely on indirect cues, such as odour or colour to predict the reward value of a flower. The hawkmoth *Manduca sexta* has been shown to innately prefer flowers emitting oxygenated aromatic compounds and certain terpenes^11^,^12^. However, even among these flowers, corolla morphology and nectar energy varies greatly^13^ and studies correlating olfactory preferences of a pollinator and the reward value of different flowers are still scarce. Interestingly, flowers not emitting volatiles have been shown to receive less pollination service by *M. sexta* than those emitting volatiles, but lacking nectar, indicating that floral scent might indeed be a more important predictor for a nectar reward, than the reward itself^14^. In those cases, in which pollinators do strongly rely on volatiles to predict the energy gain of a flower, volatiles might indeed support the establishment and stabilization of specialized plant–pollinator interactions.

Floral volatiles typical for a ‘hawkmoth pollination syndrome' are often associated with a long slender shape of the corolla, seemingly fitting the proboscis of a hawkmoth^15^. However, evidence that individual pollinators might be foraging more efficiently on flowers matching their proboscis length has rarely been presented. In a competitive situation between several bee species, each one utilizes mainly those flowers matching its proboscis, suggesting that each bee species might have a foraging advantage on its matching flower^16^. Moreover, it has been shown that long-tongued individuals sometimes have longer handling times than short-tongued individuals, indicating that simply increasing the proboscis length might not be an evolutionary stable strategy^2^,^17^. Similarly, a study on hummingbirds using artificial flowers found that birds foraging on matching flowers exhibited the shortest handling times^18^. Although these studies provide a good indication that foraging on matching flowers is energetically advantageous for a pollinator, a full energy balance on natural flowers might be required to draw further conclusions. Most studies on energy use have inferred energetic cost only indirectly, for example, by measuring foraging time^19^,^20^. Hence, the question whether the need to optimize foraging has indeed influenced the coevolution of matching proboscis and flowers, still remains controversial.

On the basis of these previous findings, we aimed to test whether the hawkmoth *M. sexta* does exhibit a behavioural preference towards volatiles emitted by flowers matching its proboscis and whether this preference is indeed adaptive for the moth.

*M. sexta* has a close association with different plant species of the *Nicotiana* genus, thereby offering an attractive system for the study of plant–pollinator interactions^21^,^22^. We selected seven *Nicotiana* species that overlap in geographic range with the distribution of *M. sexta* and are known to be moth pollinated^23^,^24^,^25^, but vary in corolla lengths (Fig. 1). Using wind tunnel assays and three-dimensional (3D) video tracking, we tested whether *M. sexta* has an innate preference for those flowers most closely matching its proboscis in length. Furthermore, we performed inflight respiration measurements and nectar calorimetry to investigate whether *M. sexta* does indeed forage most optimally on the one flower directly matching its proboscis. Taken together, our results demonstrate that *M. sexta* exhibits an innate preference for *Nicotiana* flowers matching its proboscis, and only these flowers contribute significantly to the energy gain of the moth during foraging. Through these results, we show how coadaptation mediated by floral volatiles can arise even in apparently generalized pollination systems, supporting Darwin's hypothesis on the coevolution of pollinators and flowers.

## Results

### Morphological match

**Figure 1:**
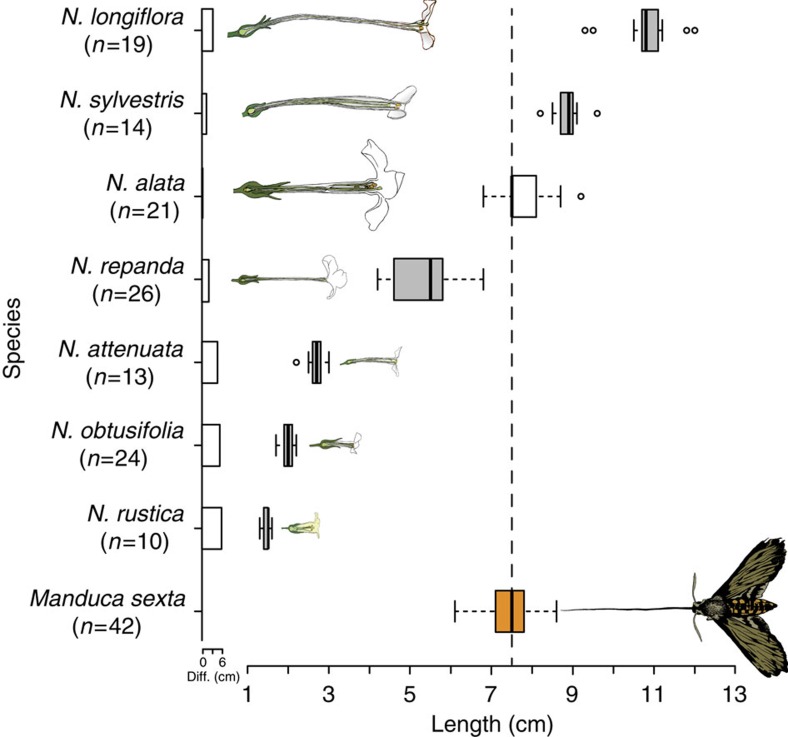
Corolla and proboscis lengths of seven *Nicotiana* species and the potential pollinator *Manduca sexta.* Grey boxes indicate corolla lengths that significantly differ from the length of the moth proboscis (orange box, dotted line indicates proboscis median length; *P*<0.05, Kruskal–Wallis test followed by Holm corrected Wilcoxon rank-sum test), while the length of the *N. alata* corolla (white box) does not differ from the proboscis length (*P*>0.05). Bar plots next to the flower names indicate absolute differences between corolla length and the moth's proboscis (cm).

The flowers of all tested *Nicotiana* species emit volatiles that can be detected by *M. sexta* and have previously been argued to be associated with moth pollination^25^,^26^ (Supplementary Fig. 1). However, while the corollas of six species are either significantly shorter (four species) or longer (two species) than the proboscis of *M. sexta*, only the corolla of *Nicotiana alata* matches the proboscis length (median *N. alata*=7.5 cm, *n*=21; median *M. sexta*=7.5 cm, *n*=42; corrected Wilcoxon rank-sum test, *P*=0.2568; Fig. 1).

### Flight behaviour and olfactory preference

**Figure 2:**
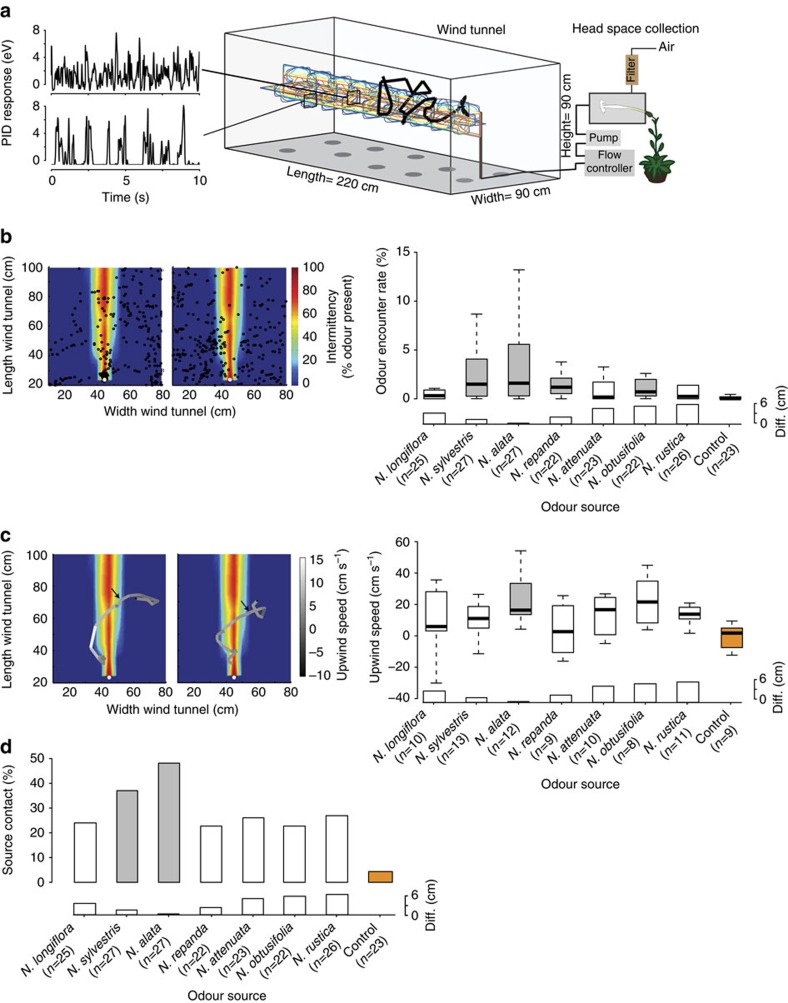
Behavioural responses of *Manduca sexta* towards headspaces of different *Nicotiana* flowers. (**a**) Experimental set-up. Bold black line, example trace of a moth approaching the odour source; coloured pattern in tunnel, representation of odour intermittency within a plume as derived from Photo-Ionization Detector (PID) measurements. Left: PID recordings in central (top panel) and peripheral (lower panel) positions of the plume. Each amplitude denotes a filament. (**b**) Moth's encounter rate with odour plumes of different *Nicotiana* species. Few but long filaments at source result in a high odour encounter rate (% of time the moth encounters the odour at a specific location in the wind tunnel) as well as short, but many filaments downstream of the source. Grey boxes, *P*<0.05 to no-flower control (Kruskal–Wallis test (*P*<0.0001) followed by corrected Wilcoxon test). Outliers not shown. Bar plots above flower names indicate absolute differences between corolla length and the moth proboscis (cm) as shown in Fig. 1. Left panel: example flight recordings with *N. alata* (left) and *N. obtusifolia* (right) as stimuli, superimposed on the reconstructed odour plume. Black circles represent the last 400 individual tracking events (tracking rate 10 Hz) of two individual flights. Colours indicate the intermittency (that is, the per cent of time odour was measured at that position in the tunnel). Light grey dots indicate position of the odour source. (**c**) Median upwind speed during 0.5 s after first contact with odour plume. Grey boxes, *P*<0.05 to no-flower control (Kruskal–Wallis test (*P*<0.0001) followed by corrected Wilcoxon test). Grey boxes, *P*<0.05 to no-flower control (Kruskal–Wallis test (*P*=0.0432) followed by corrected Wilcoxon test). Outliers not shown. Bar plot as above. Left panels: example flight tracks (*N. alata* (left) and *N. attenuata* (right) as stimuli) reconstructed from individual tracking events similar to those shown in **b** 1 s before to 2 s after plume encounter. Flight tracks are superimposed on the reconstructed odour plume. Black–white colour scale indicates upwind speed of moth (cm s^−1^). Heat map as above. Black arrow indicates the point of first plume contact. (**d**) Mean per cent of source contacts when different flower headspaces were provided. Grey colour, *P*<0.05 to no-flower control (Bonferroni-corrected Fisher's exact test).

By characterizing the odour filament distribution within the plume (Fig. 2a), using a photoionization device (200 A miniPID, Aurora) and by analysing the moths' flight patterns with a custom-built 3D tracking system, we were able to estimate the odour encounter rate for each moth and flight (Supplementary Movie 1). As the odour encounter rate was highest in the core of the plume, only animals approaching the source on a direct path would experience a high encounter rate (Fig. 2a). The quantification of odour encounters by a flying moth, hence, informs about the moth's motivation and performance to focus on the core of the plume. When we calculated the odour encounter rate per second of flight, most flowers differed significantly from the no-flower control (*n*=22–27, Kruskal–Wallis test, *P*<0.0001), with the highest odour encounter rates being observed for *N. alata* (median=1.60%, *n*=27, corrected Wilcoxon rank-sum test, *P*<0.001) and *N. sylvestris* (median=1.49%, *n*=27, corrected Wilcoxon rank-sum test, *P*<0.001; Fig. 2b). Interestingly, the longest as well as the shortest flower did not differ from the control (*N. longiflora*: median=0.32%, *n*=25, corrected Wilcoxon rank-sum test, *P*=0.9875; *N. rustica*: median=0.24%, *n*=25, corrected Wilcoxon rank-sum test, *P*=1.0).

We found such rapid upwind surges when presenting the headspace of *N. alata* to hungry moths (as compared with a no-flower control, median=16.38 cm s^−1^, *n*=12, corrected Wilcoxon rank-sum test, *P*=0.037; Fig. 2c), while none of the other tested flower odours induced significantly increased upwind speed. Finally, the flower odour plumes of *N. alata* and *N. sylvestris* were the only ones that resulted in significantly more animals, reaching the source as compared with the control (*N. alata*: median=48.15%, *n*=27, corrected Fisher's exact test, *P*=0.0043; *N. sylvestris*: median=37.04%, *n*=27, corrected Fisher's exact test, *P*=0.044). Consequently, we conclude that although most of the tested *Nicotiana* species triggered behavioural responses, only the odours of *N. alata* and to a lesser extent *N. sylvestris*, that is, those species whose corollae exhibit the best fit to the *M. sexta* proboscis, provoked behaviour that finally guided *M. sexta* moths to the source.

### Nectar amount and calorific value

**Figure 3:**
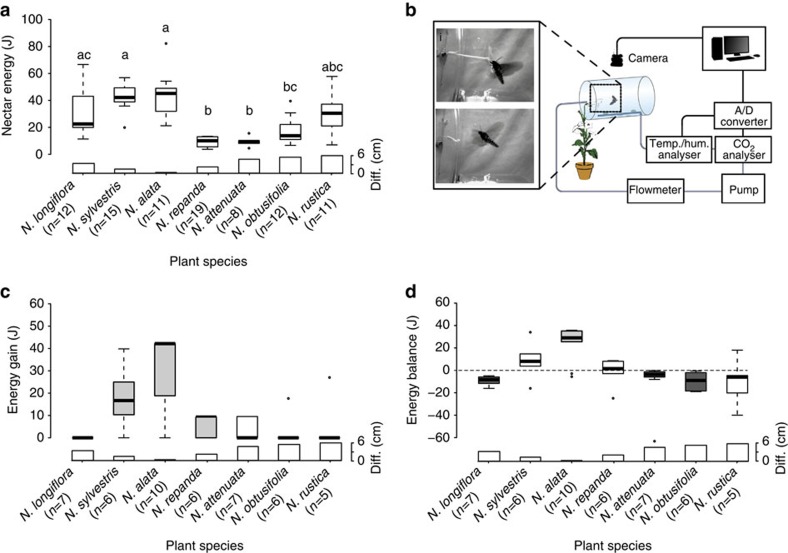
Cost–benefit analyses of *Manduca sexta* flower handling. (**a**) Total amount of energy per flower provided by the different plant species. Letters indicate significant differences (*P*<0.05, Kruskal–Wallis test (*P*<0.0001) followed by corrected Wilcoxon test). Black points indicate outliers. (**b**) Schematic drawing of the set-up used for measuring foraging efficiency. Pictures show *M. sexta* foraging on *N. alata* and below *N. obtusifolia*. (**c**) Gross-energy gain (J) of *M. sexta* when foraging on different flowers. Grey colour, *P*<0.05 (one-sided Wilcoxon test against zero). (**d**) Energy balance (J) of *M. sexta* after foraging on different *Nicotiana* plants. Light grey colour indicates flowers significantly greater than zero; dark grey indicates flowers significantly smaller than zero (*P*<0.05, two-sided Wilcoxon test against zero).

Since not nectar volume, but energy content is of ultimate importance for the moth, we analysed the calorific value of the nectar in each tested species, using a gas chromatography–mass spectrometry (GC–MS)-based approach (Fig. 3a). Although the nectar volume was related to the flower length (Supplementary Fig. 2a), the nectar of longer flowers was often less concentrated (Supplementary Fig. 2b). Therefore, the energy provided by flowers of different length was mostly similar and unrelated to flower length. Hence, nectar energy on its own did not explain the moths' foraging preference.

### Nectar gain

Knowing the nectar energy content for each *Nicotiana* species, we next analysed how much of this nectar was consumed per moth and visit. We then estimated the species-specific gross-energy gain attained per moth and flower visit (Fig. 3c). While the moths gained significant amounts of energy from visiting the flowers of the two attractive species *N. alata* (median=42.17 J, *n*=10, corrected Wilcoxon rank-sum test, *P*=0.0059) and *N. sylvestris* (median=16.68, *n*=6, corrected Wilcoxon rank-sum test, *P*=0.0359), visiting four out of five of the less attractive flowers resulted in a gross-energy gain not significantly different from zero. In case of the shorter flowers, this reduced energy gain was mainly due to a low success rate in flower handling: in case of the shortest provided flower type (*N. rustica*) only 20 per cent of the moths succeeded in collecting the nectar. Interestingly, moths easily inserted their proboscis into the non-attractive *N. longiflora.* However, due to the too long corolla of this flower species the nectar could not be reached by the moths, resulting in no energy gain whatsoever from this otherwise nectar-rich species.

### Energy balance

Although the gross-energy gain of the moth did already explain the behavioural preference of the moth to a certain degree, the choice for *N. alata* became even more understandable when we in addition took into account the energetic costs a moth faced per flower visit. To do so, we measured the respiration of the moth while foraging on different flowers, using a custom-built set-up (Fig. 3b; Supplementary Movie 2), which allowed us to measure the ambient CO_2_ increase during a single-flower approach in free flight. When we calculated the net-energy gain (that is, balanced the gross-energy gain per flower visit against the rate of energy spent), the moth's preference for *N. alata* turned out to be highly adaptive. Only feeding on flowers from *N. alata* resulted in a significantly positive net-energy gain (median=28.27 J, *n*=10, corrected Wilcoxon rank-sum test, *P*=0.014; Fig. 3d), indicating that *N. alata* would indeed be the optimal and most reliable foraging choice.

## Discussion

Pollinators and plants have coevolved with respect to certain traits such as morphology, colour or scent, leading to so-called ‘pollination syndromes'^27^. This coadaptation within multiple traits has often been argued to lead to a specific advantage for a suiting pollinator, driving the evolution of such specialist pollination systems^6^. In the present study, we show that *M. sexta* has an innate olfactory preference for those flowers whose corolla length matches the length of the moth's proboscis. When analysing foraging costs and gains of naive moths at flowers with differing corolla lengths, we found that only matching flowers significantly contributed to the moth's net-energy gain. Therefore, this study provides new evidence on how specialized pollination systems can be stabilized through physiological advantages and how this specialization is strengthened by the sensory system of the animal.

Floral volatiles are of particular importance to night-active pollinators such as *M. sexta* to effectively locate a suitable flower^28^,^29^. However, the mechanisms by which pollinators use volatiles to find and assess flowers are still far less understood than the use of visual cues in plant–pollinator interactions^9^. Flying insects following an odour plume usually navigate, using the frequency of odour encounters (that is, the percentage of time during which the moth encounters the odour) rather than the odour concentration^29^,^30^. Notably, odour filaments maintain the specific volatile composition emitted by the flower and would thereby allow the pollinator to recognise the identity of a flower also at a distance^30^. Our results highlight the importance of the odour encounter rate for *M. sexta* to navigate towards a flower in the absence of informative visual cues. Moreover, we found that already the first encounter with the odour plume did enhance the upwind movement of the moth. The extent of the upwind movement depended on the presented flower, indicating that flower-specific differences in the detection and/or valence of floral volatiles were already established at a distance of >1 m. These results are coherent with pheromone-induced flight behaviour, where a single encounter with the pheromone plume does already trigger a rapid upwind surge of a male moth^31^,^32^. *M. sexta* responds to the loss of an odour plume with casting flights, that is, zig-zag flights perpendicular to the wind direction^33^. Correspondingly, we found a fast increase in upwind speed upon plume encounter, which was sustained for ∼2 s. Rapid turns and sudden increases in flight speed are complex behavioural tasks, which involve sensory feedback from the antenna^34^ and the wings^35^. Nonetheless, the interaction between flight control and the olfactory system is not fully understood^36^, and further work might be needed to test the adaptations of *M. sexta* to track the complex odour plumes of flowers in flight.

Similarly, to pheromone communication between male and female moths, a strong preference towards certain floral volatiles could lead to the development of a ‘private channel' between a pollinator and a flower, which would then further increase coadaptation. However, such a development is often limited by phylogenetic constraints both on the evolution of olfactory genes in the moth, as well as on the development of genes regulating volatile production in the flower^25^,^37^. Most of the physiologically active compounds that were emitted by the flowers tested in our study (for example, benzyl alcohol or different aldoximes) derive from the amino acid phenylalanine^26^,^38^, indicating that many of these plants use similar pathways for the production of floral volatiles. Nevertheless, all plants also emitted unique compounds, which should allow the moth to discriminate between the different plants (Supplementary Fig. 1). Interestingly, *Petunia axillaris* another flower that has repeatedly been found to be pollinated by *M. sexta* does match the moth's proboscis in a similar way as *N. alata* and also emits a similar volatile profile^39^,^40^. In *Petunia*, the synthesis of these volatiles is mainly regulated by a single transcription factor^41^. Correspondingly, recent work on the *M. sexta* olfactory genes has revealed several lineage specific expansions^42^. These genetic duplication events might have increased the sensitivity of sphingid moths towards specific floral volatiles, parallel to what has been suggested for the *Drosophila* genus^43^. Thus ‘private channels' between pollinators and plants could evolve based on the relatively small genetic changes, which would then allow a coadaptation to take place.

Given the high spatial and temporal variability of floral resources, it has been a longstanding question under which circumstances pollinators should have developed a behavioural preference, that is, become specialized^8^. Most flowers in our study provided a similar amount of nectar energy, suggesting that the interaction between *M. sexta* and the different *Nicotiana* flowers was not determined by any flower-specific differences in nectar gain. We therefore asked, whether flower-specific foraging costs might play a role in shaping the interaction between *M. sexta* and the different plants. Foraging costs are particularly high for species feeding ‘on the wing' such as bats, hummingbirds and especially hawkmoths^3^,^44^. In addition to this, foraging costs may vary considerably depending on the nectar load or environmental conditions^45^,^46^. Hence, direct energy measurements were needed to fully determine the impact of foraging costs, especially on the behaviour of hovering pollinators. In our study, we found hovering costs ranging from 0.26 W g^−1^ for matching flowers to 0.54 W g^−1^ for too long or too short flowers. Hovering costs at flowers with a corolla lengths similar to the length of the moths' proboscis were not different from the reported energy expenditure of *M. sexta* during hovering without flower handling of 0.23 W g^−1^ (ref. 47). However, the energy consumption was significantly higher at the two shortest and the longest flowers (Supplementary Fig. 3). While high costs at the longest flower, *N. longiflora*, might have resulted from the constant attempt to reach the nectar source, high costs at the too short flowers might arise because these flowers provided less stabilization for the inserted proboscis and thereby a less stable hovering flight, which has been shown to increase the energy consumption due to a constant need for acceleration and deceleration^48^. In addition, it has to be taken into account that on the shorter flowers moths had a success rate of only 20%, which again increased foraging costs, as the moths were forced to invest several costly attempts before reaching the nectar. Together, the high hovering costs and the low success rates significantly influenced the foraging outcome of the moths when visiting the different flowers. However, we only tested naive hawkmoth during their first contact with a flower and as it has been shown that experience can improve flower handling performance^49^, one can assume that repeated contact even with the morphologically less fitting flowers could result in a positive energetic outcome. Therefore, the preference for matching flowers should not prevent the moth from visiting other flowers, in case matching flowers are absent in a habitat or so far apart that travelling costs exceed flower handling costs. Indeed, in the absence of innately preferred flowers, *M. sexta* has been shown to flexibly widen its foraging scope, but also returns to its innately preferred flower when these become again available^11^,^12^. However, as experience could also improve the handling efficiency on matching flowers, one would assume that the relative order of the flower preference and the net energy gained from the different flowers would remain also for experienced moth or would even be further strengthened, as it has been shown that the learning rate of insect pollinators also depends on the gained energy reward^50^.

As Waser *et al*.^8^ pointed out, a flower preference in pollinators becomes beneficial when travel costs are low compared with the costs for flower handling. Willmott and Ellington^46^,^51^ calculated the cost of forward flight for *M. sexta* at travelling speed (3 m s^−1^) to be 0.13 W g^−1^. Hence, different from the bumblebee where the cost of hovering and forward flight have been found to be similar^52^, hawkmoths experience 2–4.2 times higher costs while handling a flower than while travelling between flowers. Taken together, the energetic characteristics of the interaction between *M. sexta* and the different *Nicotiana* species might indeed facilitate the development and maintenance of a strong receiver bias by *Manduca* towards volatiles of matching flowers.

Our results suggest that the difference in net-energy gain of *M. sexta* obtained from different flowers has influenced the coevolution between *Nicotiana* flowers and one of their main pollinators, *M. sexta*. However, energy gain might not always translate into direct fitness advantages, since other factors such as predation and mating success might further influence the life history of an animal^53^. Aerially foraging bats, present a major predation threat to nectar-foraging moths during the search for suitable flowers^54^. A reduced searching time due to an increased detectability or valence of floral volatiles, such as we found in this study might therefore not only increase the energy gain, but could also reduce predation risk. In addition, nectar feeding in hawkmoths leads to a fivefold increase in female fecundity^55^ and it has even been suggested that carbon availability limits the life history of nectar-feeding lepidopteran to an even greater extent than nitrogen limitations^56^. Hence, behavioural preference towards flowers with a higher energy gain could indeed lead to fitness differences for hawkmoth foraging on different flowers, even though such a preference might also render this pollination system more vulnerable to environmental and climatic changes^57^.

In *On the Origin of species* Darwin wrote: ‘Thus I can understand how a flower and a bee might slowly become, either simultaneously or one after the other, modified and adapted to each other in the most perfect manner, […].', suggesting for the first time a coevolution of plants and pollinators. Our study shows that, although *M*. *sexta* might potentially forage on a variety of flowers, only those flowers matching the specific morphology of this pollinator contribute significantly to their energy gain during nectar foraging, supporting Darwin's initial hypothesis. We furthermore demonstrate that this interaction of moth and flower is mediated by the olfactory preference of the moth. Potentially, this reciprocal interaction between morphological fit and chemical communication of flowers and moths was the precursor of the evolution of numerous specialist pollination systems. Our results therefore stress the importance of chemical communication for pollination and conservation ecology.

## Methods

### Insect rearing

All animals used were reared at the Max Planck Institute for Chemical Ecology, Jena, Germany, as described in detail by Koenig *et al*.^42^ Eggs were obtained from female moths, which were kept under ambient conditions and provided with *N. attenuata* plants for oviposition. Larvae were subsequently maintained on artificial diet at 70% relative humidity and 27 °C with a light:dark cycle of 16:8. Fifth-instar caterpillars were individualized for pupation and left till 1 week before adults enclosed from the pupae at the same climate conditions. Pupae were sexed, and male and female pupae were transferred to separated flight cage with a light:dark cycle of 16:8, 70% relative humidity and 25 °C during the light phase, and 60% relative humidity and 20 °C during the dark phase. Only adult male moths between 75 and 80 h after enclosing from the pupae were used for all experiments.

### Plant breeding and headspace collection

All plant species were grown at the Max Planck Institute for Chemical Ecology, Jena, Germany, for several generations. Plants were grown in the greenhouse at 23–25 °C, 50–70% relative humidity and a light:dark cycle of 16:8 h (Philips Son-T Agro 400 W Na vapour bulbs, 350–500 μmol m^−2^ s^−1^ photosynthetic photon flux at plant level) until elongation. At least 1 week before the experiment plants were transferred to a climate chamber with the same settings as the moth flight cage. Plants were watered daily with 100 ml tap water supplemented with 0.12 g l^−1^ fertilizer (Peters Professional Allrounder, Planta Düngemittel, Germany, nutrient composition: 20% N, 20% P_2_O_2_, 20% K_2_O, 0.015% Cu, 0.12% Fe, 0.06% Mn, 0.01% Mo and 0.015% Zn). For all experiments we used flowers from 5 h till 9 h after the start of anthesis.

To provide a natural headspace of a single flower to the moth in the wind tunnel, we used a set-up as depicted in Fig. 2a. The plant was contained in a separately ventilated compartment behind the wind tunnel. Individual flowers were then carefully placed into a custom made plastic (polyoxymethylene) collection chamber through a small opening without detaching the flower from the plant. The opening was further sealed with cotton wool just behind the sepal leafs. The collection chamber had a fixed volume of 200 ml. To actively collect the floral headspace, we pushed charcoal-filtered air at a rate of 0.9 l min^−1^ into the chamber, while simultaneously pulling 0.7 l min^−1^ out of the chamber and into the wind tunnel using teflon tubing. In the wind tunnel, the plant headspace was released through a small opening (Ø 4 mm). The slight overpressure was applied to exclude contamination with green leave volatiles from the plant. All flowers were placed at least 1 h before the start of the experiments. Through this set-up, we aimed to present the most natural olfactory stimulus possible to maintain the exact blend composition and emission rate of every flower. Although a previous study using a similar set-up has highlighted the importance of the blend composition over the blend strength^58^, we cannot conclude whether the here-described preference for *N. alata* is solely based on the blend composition or also on the emission rate. Nonetheless, our data show a clear preference just based on the headspace of a single flower with its natural characteristics.

### Odour plume reconstruction

Odour distribution and pulse dynamics in the wind tunnel were measured using a Photo-Ionization Detector (200 A miniPID, Aurora Scientific, Canada) and acetone as a tracer gas. In the close vicinity of the source (0–20 cm), we measured the concentrations in steps of 5 cm along the *x*, *y* and *z* axis. With greater distances from the source, we decreased the measuring steps to 20 cm along the three axes. At each point in space we recorded for 2 min. Data acquisition and storage was done via LabVIEW (National Instruments, USA), further analyses were performed using Matlab (Mathworks, USA). To determine odour filament frequency and the percentage of odour presents, we set a threshold of 1 eV and counted the number of signals above this threshold. On the basis of these values, we derived an odour intermittency value for every point in the wind tunnel by linear interpolation.

### 3D tracking of moth flights

Hawkmoth inflight response to floral volatiles was analysed in a wind tunnel (plexiglass, 220 × 90 × 90 cm^3^) set at a constant airflow of 0.4 m s^−1^, 0.5 lux light, 25 °C and 70% humidity. Flight paths were captured using a custom-built tracking system. Initial images were recorded at 30 Hz by four cameras (Logitech), two positioned on top and two at the side of the wind tunnel. Cameras were set to a resolution of 800 × 600 pixels, with each pixel having a size of ∼0.3 cm^2^. The position of the moth was calculated at a rate of 10 Hz based on a background subtraction algorithm implemented in C. Further analyses of tracking data were performed using custom-written Matlab scripts. Individual hawkmoths were kept in small mash tubes (diameter, 13 cm; height, 15 cm) in a pre-exposure chamber at the same temperature, humidity and light as in the wind tunnel for about 1 h before the experiment. For testing, the opened mash tubes were inserted into the wind tunnel onto a take-off platform. Each moth was given 5 min to initiate wing fanning to be considered for analyses. After taking flight moths were recorded for 4 min. All experiments were performed within the last 2 h of the moth's scotophase.

### Flights within plume

By aligning the odour filament distribution within the plume with the 3D tracked flight patterns, we estimated the number of odour signal contacted for each moth and flight. As the odour signal was highest in the core of the plume, only animals approaching the source on a direct path would have encountered a high number of odour encounters. The quantification of odour signal detected by a flying moth, hence, informed about the moth's motivation and performance to focus on the core of the plume.

### Plume-induced upwind flights

We analysed the upwind speed of the moth shortly after the encounter of the odour plume. For this, the recorded flight tracks were combined with the reconstructed odour plume. We first determined the first point at which a moth based on the reconstructed plume would have encountered the odour signal above 1 eV (noise threshold), during the 4 min of free flight. Starting from this point, we calculated the mean upwind speed over the next 0.5 s, resulting in a single value for each animal. We then compared the upwind speeds of moths tested with flower plumes with those of moths tested with an empty control.

### Nectar analyses and net-energy calculation

The energetic outcome of the moth foraging flight is affected by the moth's energy invested and the energy gained through the nectar. Therefore, we investigated the nectar energy provided by the flowers of different plant species, using a GC–MS-based approach. First, we collected nectar from flowers 5–9 h after the onset of anthesis, using 3–5 plants per species. To do so, all flowers of one plant were collected and the corolla tube was cut at half length. We then placed the flower part containing the nectaries upside down in a 50-ml reaction tube and centrifuged for 2 min at 1,000 r.p.m., which caused the nectar to accumulate at the bottom of the reaction tube. Subsequently, the fresh weight of the nectar was noted and the sample was freeze-dried overnight at −80 °C and 0.014 mbar. Dried samples were weighted again and dissolved in a ratio of 1 mg:1 ml in pyridine. To increase the nectar volatility, we derivatized our samples by taking a 40 μl aliquot of the sample and adding 50 μl BSTFA (*N*,*O*-bis(trimethylsilyl)-trifluoroacetamide) supplemented with 1% TMCS (trimethylchlorosilane). In addition, 10 μl phenyl-ß-D-glucoside (Fisher Scientific) dissolved in 1 mg:1 ml pyridine was added as internal standard. The sample was then shaken for 90 min at 37 °C and 225 r.p.m., before being further diluted by adding 900 μl pyridine. After derivatization, we injected 1 μl of our sample into the GC–MS (Agilent Technologies 7890 A, Aglient, USA), using a non-polar HP-5 column (30 m, 0.25 mm ID and 0.25-μm film thickness; Agilent, USA) and operating in split-less mode, with the injection port set to 240 °C. Helium was used as a carrier gas (1.1 ml min^−1^). The program started at an initial temperature of 60 °C for 3 min and was increased by 4 °C per min to a final temperature of 300 °C. To identify and quantify the outcome of the GC–MS analyses, we used concentration curves of pure sugars (fructose, glucose and sucrose at 1, 2.5, 5, 10, 15 and 20 μl). We then calculated the total energy per mg fresh mass of nectar for each plant species by summing the amount of all sugars multiplied with their specific energy content^59^, and by multiplying this value with the dry to fresh mass ratio of each species. We calculated the mean total energy content for each species by taking the mean amount of nectar fresh mass from 20 additional flowers of each species and multiplied this value by the mean energy value of 1 mg of nectar from that species. The net energy of every foraging flight was derived by subtracting the energetic costs measured by respirometry from the mean energy content calculated for the specific flower species. For those flowers, where there was still nectar remaining in the flower after foraging, we subtracted the amount of the remaining nectar from the mean nectar value of the plant species.

### Respiration measurements

Energy expenditure by the pollinator is a crucial factor when addressing the outcome of a flower–pollinator interaction. We therefore measured the CO_2_ exhaled by *M. sexta* during flower handling via a flow-through respirometry system. Previous studies have often measured O_2_ in addition to CO_2_ to determine the substrate of the energy production by the animal^60^,^61^,^62^. However, as O_2_ is present at a high background concentration, it is often difficult to be measured accurately for small animals, such as *M. sexta* at a high temporal resolution^63^. Since our study aimed to analyse energy expenditure at a relatively high temporal resolution, we have focus on measuring CO_2_ emissions. The system was set up within a fully controllable climate chamber and consisted of a sealed glass cylinder (49 × Ø34 cm) from which the air was pumped into a closed loop through a non-dispersive infrared analyser (Li-820, Licor GmbH, Germany) and back into the cylinder at a rate of 2 l min^−1^. Since temperature and humidity might influence the CO_2_ measurements, we simultaneously recorded these two parameters in the air stream ahead of the CO_2_ measurement (Sensirion SHT 75, Switzerland). Both sensors were connected to a PC outside the chamber and operated via LabVIEW (National Instruments, USA). Before every experiment, we placed an intact flower inside the chamber in such a way that the flower remained attached to the plant, but no leaf tissue was enclosed. Previous studies on *Datura wrigthii* flowers have shown that these flowers emit considerable amounts of CO_2_ directly after opening, which then decline until ∼4 h after anthesis^64^. To exclude any influence of the flower respiration on our measurements, we used only flowers 5–9 h after anthesis and analysed the emissions of several single flowers of each species and a resting moth. However, these measurements did not show any CO_2_ emissions detectable by our system (Supplementary Fig. 4). After setting up the system in this way, we allowed the open cylinder and the surrounding air to equilibrate. As soon as the CO_2_ concentration within the chamber remained constant, a moth was placed into the cylinder and the front was resealed. The moth was then allowed 5 min to initiate wing fanning and 4 min to approach the flower. During these periods the moth behaviour was constantly monitored using two cameras (Logitech C615, USA), at a resolution of 800 × 600 pixel and 30 frames per second (FPS). Videos were streamed and recorded using Noldus Media Recorder (Noldus, The Netherlands). Animals, which did not start wing fanning within this time limits, were regarded as non-responders and excluded from the statistical analyses. In case the moth fed from the flower before the 4 min had elapsed, the measurement was stopped as soon as the moth had left the flower. Hence, we recorded CO_2_ production of each moth during a single flower visit. Directly after the experiment the length of the moth proboscis was measured. The length of the corolla tube for each flower was measured as the distance between corolla base and corolla disc. To check for the amount of remaining nectar, the base of the flower was carefully opened, the nectar was sucked out using a capillary and the amount of nectar was determined with an electronic balance. The remaining nectar amount in visited flowers was compared with the amount of nectar in non-visited flowers (see above) to calculate the nectar uptake per visit.

### Volatile collection

For volatile collection, we used a push–pull system modified from Kessler *et al*.^21^ Individual flowers were carefully placed into a custom made plastic (polyoxymethylene) collection chamber through a small opening without detaching the flower from the plant. The opening was further sealed with cotton wool just behind the sepal leafs. The collection chamber had a fixed volume of 200 ml. To actively collect the floral headspace, we pushed charcoal-filtered air at a rate of 0.5 l min^−1^ into the chamber, while simultaneously pulling 0.4 l min^−1^ through a glass tube (ARS, USA); packed with glass wool and 20 mg of Super Q (Alltech, Germany). The slight overpressure was applied to ensure that no leaf volatiles would be collected. All volatiles collections using SuperQ filters lasted for one full scotophase (8 h). Finally, volatiles were eluted rinsing SuperQ filters three times with 100 μl dichloromethane(DCM). Samples were then stored at −20 °C till further analyses. For each plant species, we sampled 3–5 flowers from different plant individuals.

Previous to each volatile sampling, the collection chamber and tube connectors containing plastic parts were soaked overnight in Labosol (neoLab, Germany). All parts were then rinsed with distilled water and ethanol, before heating them at 55 °C for 2 h. Holders for SuperQ filters were custom made from polyether ether ketone (PEEK) and always washed with DCM, and subsequently heated at 200 °C for 2 h.

SuperQ filters were washed shortly before use in a series of methanol, chloroform, acetone, DCM and hexane. PDMS tubes were rinsed in ethanol and heated for 1 h at 200 °C under a constant flow of nitrogen.

### Volatile analyses

Collected headspace volatiles were analysed using a gas chromatograph-coupled mass spectrometer (Agilent 6890 GC & 5975C MS, Agilent, USA). The GC was used with a non-polar HP-5 column (30 m, 0.25 mm ID and 0.25-μm film thickness; Agilent, USA), operating in split-less mode at a constant flow of 1.1 ml min^−1^ with helium as carrier gas. The inlet port was set to a temperature of 240 °C and injection volume of 1 μl. The GC oven was set to an initial temperature of 50 °C, which was held for 2 min. Thereafter, the temperature was increased at a rate of 13 °C min^−1^ to 250 °C, which was again held for 5 min. The MS transfer line was maintained at 280 °C and the MS operated in electron impact mode (70 eV, ion source: 230 °C, quadrupole: 150 °C, mass scan range: 33–350 *m*/*z*, scanning rate 4.42 scan per s). Compounds were identified by comparing mass spectra against synthetic standards and NIST 2.0 library matches.

### Electrophysiology

Gas chromatography coupled with electro-antennographic detection (GC–EAD) was used to identify those compounds in the volatile blends of the flowers that are perceived by the moth antenna. For this, we clipped the antenna of a 3-day-old male *Manduca* directly above the scapulum and before the third last flagellum. The two tips of the cut antenna were then inserted into two-glass electrodes filled with haemolymph–ringer. EAD signals were recorded via Ag–AgCl and pre-amplified (10 ×) by a probe connected to a high-impedance d.c. amplifier (EAG-probe, Syntech, The Netherlands). The signals were fed into an analog/digital converter (IDAC-4, USB, Syntech, The Netherlands) and transferred to a PC. GC stimulation was done by injecting 1 μl of the sample into the GC (Agilent 6890, HP-5 column, 30 m, 0.25 mm ID and 0.25-μm film thickness; Agilent, USA). The inlet port was set to 250 °C; the initial oven temperature of 50 °C was raised by 13 °C min^−1^ to a maximum of 250 °C that was held for 5 min. The gas stream leaving the GC was split 1:1 by a 4-arm effluent splitter (Gerstel, Germany), using N_2_ (30.3 kPa) as a compensatory gas. One part of the gas stream was directed to the flame ion detector of the GC, whereas the other part was inserted into a humidified air stream (200 ml min^−1^) leading to the antenna preparation. Data from the flame ion detector and EAD were visualized and recorded simultaneously, using Syntech GC/EAD32 Software (Version 4.6; Supplementary Fig. 1). For further analyses, we exported the data in ASCII format. Voltage amplitudes were determined manually using Matlab (MathWorks).

### Data availability

The authors declare that all data supporting the findings of this study are available within the article and its Supplementary Information files.

## Additional information

**How to cite this article:** Haverkamp, A. *et al*. Innate olfactory preferences for flowers matching proboscis length ensure optimal energy gain in a hawkmoth. *Nat. Commun.* 7:11644 doi: 10.1038/ncomms11644 (2016).

## Supplementary Material

Supplementary Information Supplementary Figures 1-4 and Supplementary Reference

Supplementary Movie 1 3-d track of a moth flying in the wind tunnel. Odour source (Head space of a flower of *Nicotiana alata*) located left in the tunnel.

Supplementary Movie 2 Accumulative CO_2_ concentration in a test chamber, while a moth is foraging at a flower of *Nicotiana alata*.
