# Supplementary material for: Innate olfactory preferences for flowers matching proboscis length ensure optimal energy gain in a hawkmoth
Source: Nat Commun. 2016 May 13;7:11644. doi: 10.1038/ncomms11644 (PMC4869250; doi:10.1038/ncomms11644)
Supplement: Supplementary Information — Supplementary Figures 1-4 and Supplementary Reference [file ncomms11644-s1.pdf]

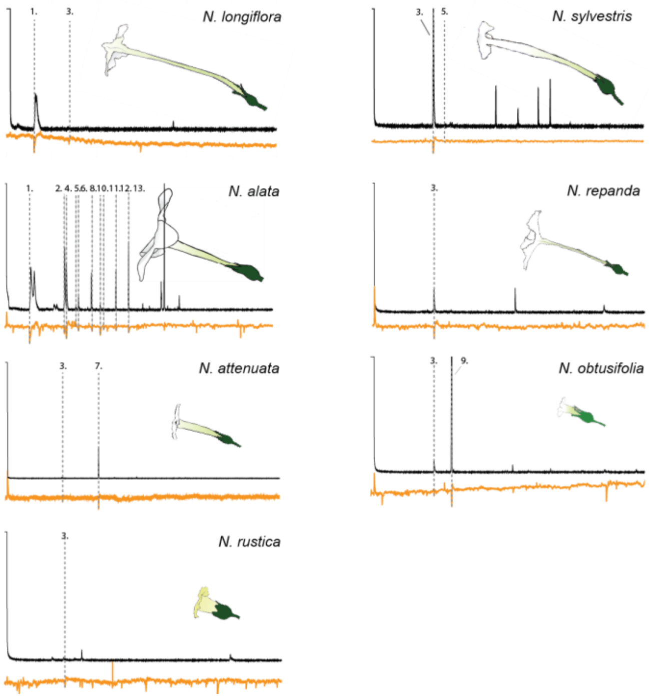

Supplementary Figure 1| Physiological response of *Manduca* towards volatiles from the tested *Nicotiana* species.

Traces show representative FID traces (blue) and corresponding EAD responses (yellow) for every flower species. Numbers depict the following compounds 1: 2 and 3- Methylbutyl aldoxime; 2: Eucalyptol; 3: Benzyl alcohol 4: Benzeneacetaldehyde; 5: Linalool; 6: Phenylethyl alcohol; 7: Unknown; 8: Methylsalicylate; 9: Geraniol; 10: 4-Methylbenzaldehyde; 11: Eugenol; 12. Isoeugenol. Each compound was verified by coelution with a synthetic standard.

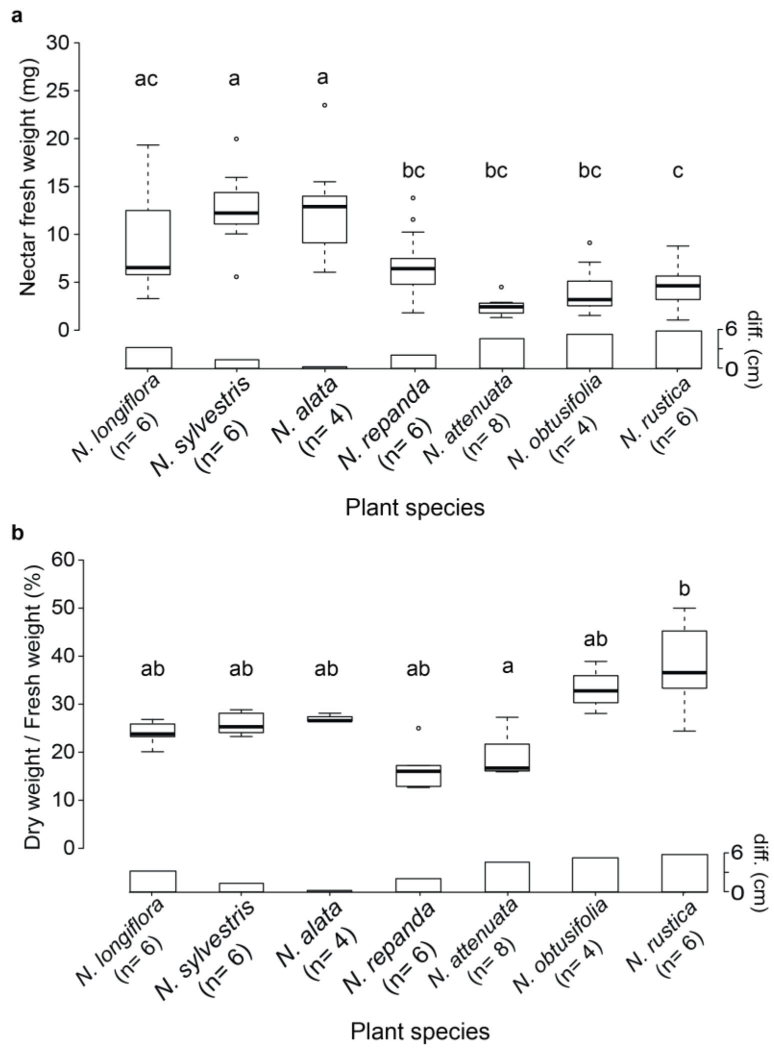

Supplementary Figure 2| Nectar amount and dry by fresh weight ratio of different *Nicotiana* species.

(a) Boxplot shows total amount of nectar (mg) per flower provided by the different plant species. Letters indicate significant differences ($P< 0.05$) according to Kruskal- Wallis test ($P<0.0001$) followed by Wilcoxon rank sum test with Holm correction for multiple comparisons. Black points indicate outliers. (b) Dry weight by fresh weight ratio shown as boxplot for the tested *Nicotiana* species. Letters indicate significant differences ($P< 0.05$) according to Kruskal-Wallis test ($P<0.0001$) followed by Wilcoxon rank sum test with Holm correction for multiple comparisons. Circles indicate outliers.

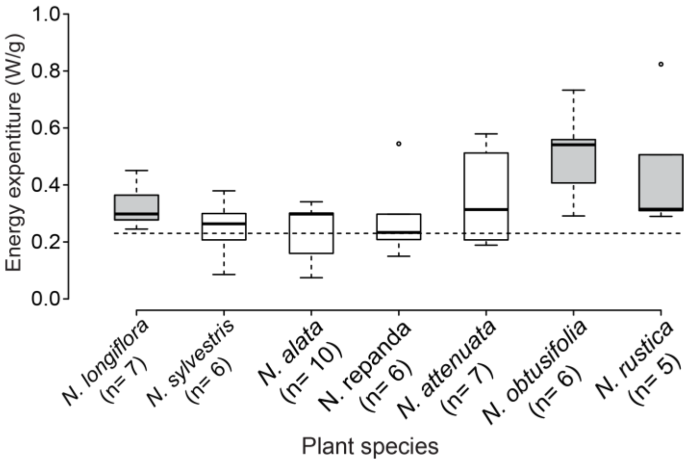

Supplementary Figure 3| Energy expenditure during foraging

Boxplot indicates energy expenditure (W/g) for *Manduca sexta* foraging on different *Nicotiana* species. Dotted line indicates energy expenditure hovering *Manduca* without flower contact [1]. Grey colour indicates energy expenditures significantly greater (*P*<0.05) than those of hovering moth without flower contact according to Wilcoxon rank sum test. Circles indicate outliers.

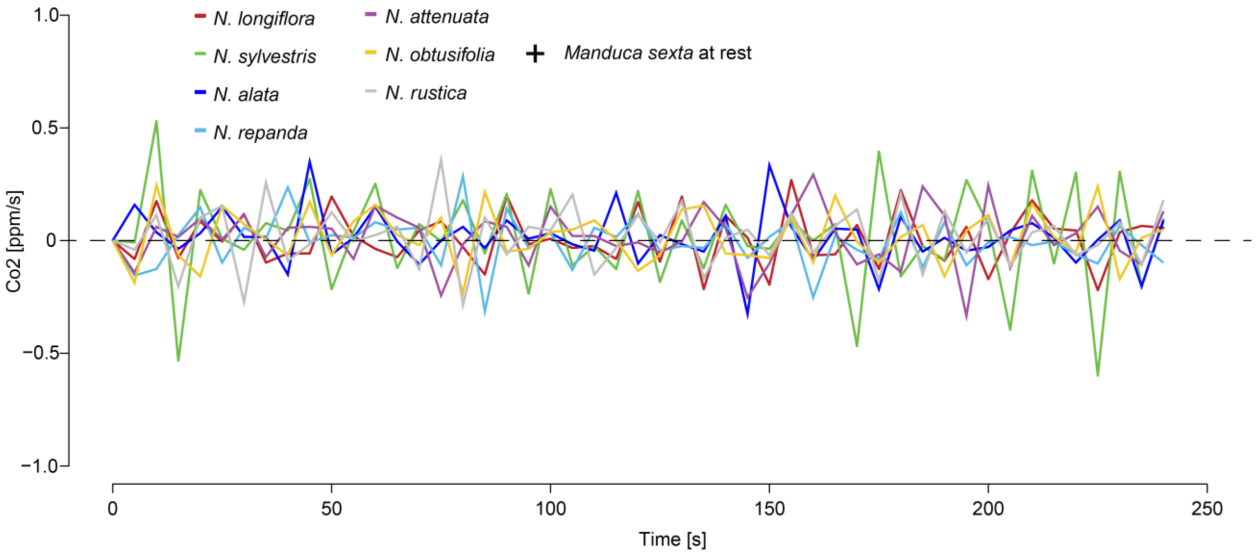

Supplementary Figure 4| Single flowers and resting moth did not result in a detectable CO2 emission rate. Different lines represent CO_2 emission rates [ppm/s] of single flowers and a moth at rest simultaneously enclosed in the respiration chamber for 4 min.

Supplementary References

1. Casey, T. M. Flight energetics of sphinx moths: power input during hovering flight. *J. Exp. Biol.* **64,** 529–543 (1976).
